# S1P Lyase Regulation of Thymic Egress and Oncogenic Inflammatory Signaling

**DOI:** 10.1155/2017/7685142

**Published:** 2017-12-03

**Authors:** Ashok Kumar, Jesus Zamora-Pineda, Emilie Degagné, Julie D. Saba

**Affiliations:** ^1^Department of Biochemistry, All India Institute of Medical Sciences (AIIMS) Bhopal, Madhya Pradesh 462020, India; ^2^Children's Hospital Oakland Research Institute and UCSF Benioff Children's Hospital Oakland, 5700 Martin Luther King Jr. Way, Oakland, CA 94609, USA

## Abstract

Sphingosine-1-phosphate (S1P) is a potent lipid signaling molecule that regulates pleiotropic biological functions including cell migration, survival, angiogenesis, immune cell trafficking, inflammation, and carcinogenesis. It acts as a ligand for a family of cell surface receptors. S1P concentrations are high in blood and lymph but low in tissues, especially the thymus and lymphoid organs. S1P chemotactic gradients are essential for lymphocyte egress and other aspects of physiological cell trafficking. S1P is irreversibly degraded by S1P lyase (SPL). SPL regulates lymphocyte trafficking, inflammation and other physiological and pathological processes. For example, SPL located in thymic dendritic cells acts as a metabolic gatekeeper that controls the normal egress of mature T lymphocytes from the thymus into the circulation, whereas SPL deficiency in gut epithelial cells promotes colitis and colitis-associated carcinogenesis (CAC). Recently, we identified a complex syndrome comprised of nephrosis, adrenal insufficiency, and immunological defects caused by inherited mutations in human *SGPL1*, the gene encoding SPL. In the present article, we review current evidence supporting the role of SPL in thymic egress, inflammation, and cancer. Lastly, we summarize recent progress in understanding other SPL functions, its role in inherited disease, and SPL targeting for therapeutic purposes.

## 1. Introduction

**Figure 1:**
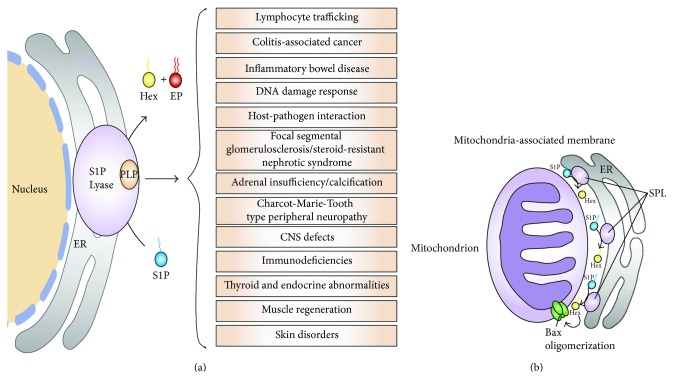
(a) SPL and its related diseases. (b) Besides endoplasmic reticulum, SPL is also localized in mitochondria-associated membrane (MAM). SPL present in MAM generates hexadecenal (Hex), which induces Bax activation.

Sphingosine-1-phosphate (S1P) is a sphingolipid metabolite and a potent signaling molecule that regulates diverse cellular functions such as cell proliferation, differentiation, and migration as well as complex processes including development, vascular maturation, angiogenesis, immune function, and inflammation [1–3]. S1P mediates most of its biological actions by binding to five S1P receptors (S1PRs) that belong to the family of G protein-coupled receptors [3]. Intracellular and circulatory S1P levels are tightly regulated by its synthesis and catabolism. S1P is generated by the phosphorylation of the long-chain base sphingosine, a step catalyzed by sphingosine kinase [4]. The resulting S1P product can be dephosphorylated by various lipid phosphatases [5, 6]. In addition, S1P is irreversibly degraded by S1P lyase (SPL), which cleaves S1P at the C2-3 carbon-carbon bond, yielding two products, ethanolamine phosphate and the long-chain aldehyde *trans*-2-hexadecenal [7–9]. Cleavage of S1P by SPL represents the final step in the sphingolipid catabolic pathway. When this step is blocked, there is no alternative pathway for sphingolipid degradation to occur. SPL depletes S1P in cellular, vascular, and tissue compartments and regulates steady-state S1P levels. Thus, SPL controls S1P pools available for interactions with intracellular targets and for autocrine and paracrine receptor-mediated signaling [9]. SPL regulates many physiological and pathological phenomena including maintenance of pluripotency in embryonic stem cells, DNA damage response, chemoresistance, muscle regeneration, satellite cell activation, thymic egress, glomerular function, adrenal function, atherosclerosis, ischemia-reperfusion injury, experimental autoimmune encephalomyelitis (EAE), central and peripheral nervous system functions, inflammation, and carcinogenesis (Figure 1(a)) [9–21]. The majority of these effects can be attributed to the ability of SPL to regulate S1P receptor signaling. However, it should be noted that some of SPL's activities may be associated with the regulation of intracellular S1P effects and with effects mediated by its products or upstream sphingolipid intermediates [22–25].

The immunological role of S1P/S1PR1, and specifically its regulation of T cell egress from the thymus and lymphoid organs, is the most well-characterized function of the S1P signaling pathway. S1PR1 expression on the surface of T lymphocytes and the presence of an S1P chemical gradient between lymphoid tissues and blood/lymph are the two essential conditions needed for lymphocyte egress from the thymus and secondary lymphoid organs [26]. Either antagonism of S1PR1 by pharmacological agents such as FTY720 or disruption of S1P gradients through SPL inhibition by 2-acetyl-4-(tetrahydroxybutyl) imidazole (THI) or its analogs block the exit of lymphocytes from the thymus and lymphoid organs, leading to lymphopenia (a reduction in absolute lymphocyte counts in the blood) [14, 27]. By preventing T lymphocytes with self-reactive receptors from entering target tissues, pharmacological agents targeting the S1P/S1PR1 axis have shown efficacy in the treatment of autoimmune diseases including multiple sclerosis, rheumatoid arthritis, and inflammatory bowel disease (IBD) [28, 29]. Although regulating T cell egress is its most well-studied immunological function, S1P also regulates other immune functions including migration of B cells, neutrophils, macrophages, natural killer (NK) cells, and hematopoietic stem cells (HSCs). Furthermore, the development and differentiation of leukocytes, mast cell degranulation, and antigen presenting cell functions are also influenced by S1P signaling [30, 31].

A link between inflammation and carcinogenesis has been appreciated for over a century [32]. It has been suggested that inflammation can promote carcinogenesis by augmenting metastasis, angiogenesis, metabolic changes favorable to cancer cells, and resistance to chemotherapy [33, 34]. S1P signaling has been shown to activate two critical transcription factors, nuclear factor kappa B (NF*κ*B) and signal transducer and activator of transcription 3 (STAT3). Both of these transcription factors regulate the transcription of large sets of genes involved in inflammation, proliferation, and carcinogenesis. Recent studies have demonstrated the role of S1P and SPL in mediating the transition from inflammation to carcinogenesis via these signaling hubs, specifically in the context of IBD and the associated phenomenon of colitis-associated cancer (CAC).

The current review is focused on the role of SPL in regulating thymic egress and procarcinogenic inflammatory signaling. We also summarize important new insights regarding S1P metabolism, SPL structure, and recently recognized functions of SPL in the context of embryonic development and the pathophysiology of human disease. We also discuss progress in the development of SPL inhibitors and their potential therapeutic applications.

## 2. Biosynthesis and Catabolism of S1P

**Figure 2:**
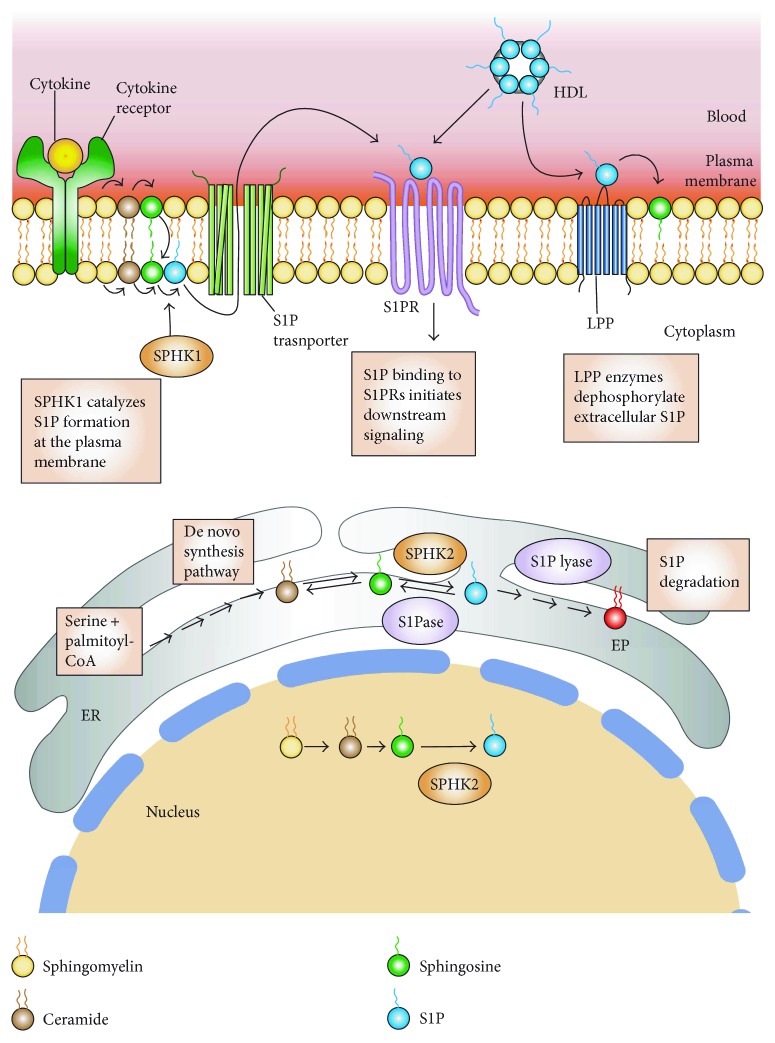
S1P metabolism, synthesis, and inside-out signaling. Sphingosine-1-phosphate (S1P) is irreversibly degraded by S1P lyase into ethanolamine phosphate (EP) and *trans*-2-hexadecenal (Hex) and dephosphorylated to sphingosine by S1P phosphatase (S1Pase) in the endoplasmic reticulum (ER) and by nonspecific lipid phosphate phosphatases (LPP) in the plasma membrane. Extracellular sphingosine formed from S1P by LPPs can be taken up by cells and be converted back to S1P or other sphingolipids. In the blood, S1P is produced primarily by erythrocytes and transported bound to albumin and high-density lipoprotein (HDL). Extracellular S1P can activate S1P receptors (S1PRs). S1P is synthesized by sphingosine kinase 1 (SPHK1) in the plasma membrane by phosphorylating sphingosine and by SPHK2 at the ER and nucleus. S1P is produced in the plasma membrane in response to stimuli and is released by specific transporters to the extracellular space where it can bind to S1PRs and initiate downstream signaling pathways (inside-out signaling).

S1P is synthesized by two highly homologous sphingosine kinases, known as sphingosine kinase 1 (SphK1) and sphingosine kinase 2 (SphK2) [35]. SphK1 is mainly localized in the cytosol, whereas SphK2 is localized in the nucleus and mitochondria-associated outer membrane (Figure 2) [25, 36]. S1P levels in the tissues, blood, and lymph are tightly regulated by six catabolic enzymes. Two S1P-specific phosphatases, namely, S1P phosphatase-1 (SGPP1) and S1P phosphatase-2 (SGPP2), are located in the endoplasmic reticulum (ER) dephosphorylate S1P, regenerating sphingosine [37, 38]. Three plasma membrane-bound lipid phosphate phosphatases (LPP1–3) [5, 39–41] dephosphorylate a broad range of lipid phosphate substrates including S1P and ceramide-1-phosphate [27, 42]. Although the LPPs do degrade a variety of lipid phosphate substrates when assayed in vitro, there does appear to be some specificity. LPP3 is more active against S1P or FTY720P than LPP1 and LPP2 [43–45]. Extracellular sphingosine formed from S1P by LPPs can be taken up by cells and be converted back to S1P or other sphingolipids. In contrast to the actions of the lipid phosphatases, which regenerate the substrate for SphK enzymes and thereby allow for reformation of S1P, the intracellular enzyme SPL catalyzes the irreversible degradation of S1P into ethanolamine phosphate and *trans*-2-hexadecenal (Figure 2) [9]. This distinction makes SPL a key factor in regulating S1P levels and chemotactic gradients, as described below.

## 3. Structure and Functions of SPL

### 3.1. SPL Structure and Mechanism of Action

SPL is a pyridoxal 5′-phosphate- (PLP-) dependent enzyme that belongs to the family of carbon-carbon lyase family of aldehyde-lyases (EC 4.1.2.27). SPL cleaves S1P between carbon atoms 2 and 3, generating *trans*-2-hexadecenal and ethanolamine phosphate [46]. SPL demonstrates a high degree of stereospecificity towards its substrates, cleaving naturally occurring *D-erythro* (2D, 3D configuration) long-chain base phosphates. However, it is not specific for chain length, degree of unsaturation, and branching of hydrocarbon chain. It can cleave S1P, dihydro-S1P, phyto-S1P, methyl-S1P, and likely also the phosphorylated form of sphingadienes, unique sphingoid bases containing two double bonds [8, 47, 48].

The SPL gene was first identified in budding yeast and named *DPL1* (for dihydrosphingosine phosphate lyase, one of the natural substrates formed in yeast cells) [49]. Subsequently, homologs have been reported in many species including mammals, insects, protozoa, bacteria, and plants [7, 8, 22, 50–56]. *SGPL1*, the human SPL gene, encodes a protein consisting of 568 amino acids with a predicted molecular weight of 63.5 kDa [8]. Human SPL shows 84% identity and 91% similarity in amino acid sequence with its mouse homolog [57]. Consistent with the high level of sequence homology among eukaryotic SPL proteins, all homologs that have been tested are capable of complementing a yeast *dpl1* mutant strain in synthetic lethal screens and other functional assays.

Human SPL predominantly resides in the ER [58]. It has also been reported to exist in the mitochondria-associated membrane [25]. The N-terminus of the SPL protein is situated in the ER lumen, whereas its active site is exposed to the cytosol [59]. Mammalian and budding yeast SPL are single-pass transmembrane ER resident proteins. Bourquin and colleagues resolved the structure of a bacterial SPL (StSPL) from *Symbiobacterium thermophilum* as well as a truncated form of DPL1 [56]. Based on the crystal structure of DPL1 and StSPL, they proposed a mechanism of S1P cleavage by the SPL-PLP holoenzyme that involves the transient formation of a PLP-S1P adduct [46, 56]. SPL enzymes, DPL1 (yeast), and StSPL (bacteria) function as a dimer [56]. In contrast to DPL1, StSPL lacks a transmembrane domain, and recombinant StSPL is active *in vitro* and *in vivo* as StSPL has been shown to cleave S1P present in cell culture medium and blood [60].

### 3.2. Tissue Distribution of SPL

Mammalian SPL is expressed in many tissues, as shown by analysis of gene and protein expression surveys. To further investigate the tissue distribution of SPL, *SGPL1* reporter mice expressing LacZ under the control of the *SGPL1* promoter were generated [61]. *β*-Galactosidase staining of whole organs and tissue sections revealed that SPL is highly expressed in the thymus, liver, stomach, jejunum, ileum, cecum, colon, brain, spinal cord, trigeminal nerve ganglion, kidney, bladder, skin, preputial gland, Harderian gland, pituitary gland, ribcage, brown adipose tissue, adrenal cortex, ovary, and testis [61]. In contrast, SPL is faintly expressed in the tongue, esophagus, and duodenum, pancreas, heart atrium and ventricle, spleen, quadriceps muscle, sciatic nerve, lung, trachea, aorta, diaphragm, mammary gland, lacrimal gland, mesenteric adipose tissue, penis, prostate, and vesicular gland [61]. SPL is also expressed in the immune cells and secondary lymphoid organs. In the spleen, SPL is expressed in both splenocytes and stromal cell fractions. However, in the thymus, SPL is strongly expressed in thymic stromal cells, whereas its expression in thymocytes is barely detectible. SPL is also expressed in circulating leukocytes, including B and T lymphocytes, monocytes, and granulocytes [61]. Erythrocytes and platelets lack SPL expression [62, 63].

SPL expression begins during early developmental stages including in the neural tube and developing brain, Rathke's pouch, first brachial arch, third brachial arch, optic stalk, midgut loops, and lung buds. Strong SPL expression was observed at embryonic day 18 in the nasal epithelium, intestinal epithelium, skin, cartilage, thymus, and pituitary gland [64]. The ubiquitous expression pattern of SPL suggests it plays an important role in the function of many types of cells and tissues. In leukocytes, the main function of SPL is likely the control of cell trafficking through S1P signaling. Similarly, SPL may be important for cell migrations that occur during development. However, SPL likely has other functions. High expression of SPL in certain tissues has been linked to their rapid cell turnover. This is exemplified by the high SPL expression observed in epithelial cells of small intestine and in the mucosal cells of the olfactory bulb, both of which exhibit a high turnover rate [65, 66]. In other tissues, high levels of SPL activity may be required to accommodate active sphingolipid recycling such as in the brain and skin, to generate products essential for organ function, or to control calcium homeostasis or cholesterol trafficking [67].

### 3.3. SPL in Development and Disease

In simple metazoans such as fruit flies and nematodes, SPL plays a critical role in the development [50, 51] (reviewed in [57, 66]). Mice that completely lack SPL activity due to targeted disruption of *SGPL1* fail to thrive and do not survive beyond the weaning period, exhibiting impaired lymphocyte and neutrophil trafficking, elevated cytokines and serum lipids, increased lipid storage in the liver, and deficient adipose stores [68, 69]. *SGPL1* null mice also develop myeloid cell hyperplasia and significant lesions in the heart, lung, bone, and urinary tract to variable degrees [70]. Humanized *SGPL1* knock-in mice exhibit 10–20% of SPL enzyme activity compared to wild-type mice. This partial restoration of SPL activity is sufficient to protect humanized SPL mice from the lethal nonlymphoid lesions that develop in *SGPL1* null mice [70]. However, humanized SPL mice remain lymphopenic, which suggests that lymphocyte trafficking is exquisitely sensitive to alteration in the S1P levels in the thymus and lymphoid organs [70].

There is evidence to suggest that a dynamic balance between S1P and ceramide is maintained within the cells, contributing to the determination of cell fate in response to stress. SPL has the ability to promote cell death by attenuating the cell survival and proliferation signals mediated by S1P [42]. SPL plays a role in stress responses [71]. Overexpression of SPL in several malignant and nonmalignant cells has been shown to sensitize these cells to DNA-damaging drugs [11, 12]. Conversely, SPL-deficient cells exhibit resistance to nutrient deprivation, heat shock, chemotherapeutic drugs, and radiation [12, 72–75].

Consistent with a role for S1P in carcinogenesis, SPL expression is altered in a number of cancers. SPL expression and enzyme activity are downregulated during intestinal tumorigenesis in APC^Min/+^ mice and in tumors from colon cancer patients [11]. While this may be an indirect result of the dedifferentiation of neoplastic tissues that normally express high SPL levels, it nonetheless influences local S1P levels and can thereby promote inflammation and carcinogenesis as described below. Downregulation of SPL expression has also been reported in prostate cancer and oral squamous cell carcinoma (OSCC) [74, 76]. In contrast, upregulation of *SGPL1* mRNA has been reported in OSCC, hepatocellular carcinoma, and ovarian cancer [77–79]. The etiology of this finding and its impact on carcinogenesis remain to be clarified.

S1P serves as a muscle trophic factor that enables efficient muscle regeneration. SPL is dynamically upregulated in skeletal muscle after injury but is otherwise undetectable in resting skeletal muscle [13]. We have further shown that S1P activates quiescent satellite cells (SC) via an S1PR2/STAT3/Rac1-dependent pathway, thereby facilitating skeletal muscle regeneration [13]. Upregulation of SPL and a decrease in S1P have also been observed in the skeletal muscle of *mdx* mice, a model for muscular dystrophy. Administration of THI to mice through drinking water raised skeletal muscle S1P levels, enhanced SC recruitment, and improved *mdx* skeletal muscle regeneration [13].

SPL has been implicated in various lung diseases such as acute lung injury, pulmonary fibrosis, pulmonary arterial hypertension, and cystic fibrosis [80–82]. Suppression of SPL activity by THI reduces lipopolysaccharide-induced lung injury and inflammation in mice [82]. SPL deficiency in the hematopoietic compartment leads to monocytosis, impaired chemokine-induced monocyte trafficking, and monocyte differentiation into macrophages with a proinflammatory phenotype. Collectively, these effects result in an attenuated atherogenic response in low-density lipoprotein (LDL) receptor knockout (KO) mice with hematopoietic SPL deficiency [19]. S1P has been shown to promote cardiomyocyte survival and contribute to ischemic preconditioning. SPL activity is increased in heart tissue after ischemia, and mice heterozygous for an *SGPL1* null allele exhibit reduced infarct size and increased functional recovery compared to wild-type mice [15]. Billich et al. generated inducible *SGPL1* KO mice exhibiting partial reduction of SPL activity. These mice are protected from neurological injury in the EAE model of multiple sclerosis, and the finding was associated with reduced T cell immigration into the central nervous system (CNS) [18].

SPL also plays an important function in host-pathogen interactions. *Legionella pneumophila* is an intracellular pathogen that can cause severe pneumonia in humans. In *L. pneumophila*, SPL (LpSpl) has been identified as an effector protein that is translocated into the host cell by the pathogen's Dot/Icm type IV secretion system. LpSpl targets the host sphingolipid metabolic pathway and reduces sphingosine levels leading to inhibition of macrophage autophagy [53, 83]. Recently, two secretory isoforms of SPL were identified and characterized in *Burkholderia pseudomallei* and *Burkholderia thailandensis*, facultative intracellular bacteria. SPL-deficient mutants of *Burkholderia* sp. displayed impaired intracellular replication in murine macrophages [84, 85]. The findings suggest that the bacterium secretes SPL to remove host-generated intracellular S1P, which somehow facilitates its survival and replication.

Recently, inherited recessive mutations in *SGPL1* have been linked to human diseases representing unique inborn errors of metabolism in four separate studies [20, 21, 86, 87]. The disease-causing mutations are truncating, nonsense mutations or missense mutations in highly conserved coding regions associated with cofactor binding and catalytic activity. Lovric et al. identified nine different recessive mutations in patients affected with steroid-resistant nephrotic syndrome (SRNS) [86]. All the detected mutations led to reduced or undetectable levels of SPL protein and undetectable enzyme activity in the affected patients' fibroblasts. *SGPL1* mutations were associated with SRNS and facultative ichthyosis, adrenal insufficiency, immunodeficiency, and neurological defects [86]. Janecke et al. identified two homozygous truncating mutations in association with congenital nephrotic syndrome, adrenal calcifications, and hypogonadism [20]. Prasad et al. identified four homozygous *SGPL1* mutations that were associated with SRNS, ichthyosis, primary hypothyroidism, neurological symptoms, and cryptorchidism [21]. In a separate study, mutations in *SGPL1* were found in association with two patients presenting with an atypical form of axonal peripheral neuropathy [87]. *SGPL1* knockout mice have been shown to recapitulate the main characteristics of the human disease with abnormal adrenal and renal morphology [21]. Vascular alterations were present in a patient's renal biopsy, in line with changes seen in *SGPL1* knockout mice that are compatible with a developmental defect in vascular maturation, a classical function of S1P [20]. Podocytes are known to exhibit S1PRs, and as components of the glomerular filtration barrier, they could exhibit increased sensitivity to circulating S1P levels, especially under conditions of reduced SPL activity. Two recent reports from the van Echten-Deckert laboratory also point to complex mechanisms by which *SGPL1* disruption influences CNS biology, one involving a product of the reaction (ethanolamine phosphate) affecting autophagy in neurons and another showing SPL loss which causes S1P accumulation, leading to calcium changes in neurons thereby inducing ubiquitin proteasome-mediated effects. These studies show the many ways in which disruption of the final enzyme of sphingolipid metabolism can cause phenotypes [88, 89].

### 3.4. Functions of SPL Products

Although most of the functions and phenotypes associated with SPL and its deficiency have been attributed to its regulation of S1P levels, there is evidence that the products of the reaction catalyzed by SPL may have additional biological functions [24]. For example, these products have been shown to promote cell proliferation through an S1P-independent pathway in F9 embryonal carcinoma cells [90]. *Trans*-2-hexadecenal induces cytoskeletal reorganization and modulates histone acetylation pattern thereby inducing the transcription of inflammatory cytokines [23, 24]. Furthermore, trans-2-hexadecenal reacts readily with deoxyguanosine and DNA to produce the diastereomeric cyclic 1,N(2)-deoxyguanosine adducts 3-(2-deoxy-*β*-d-erythro-pentofuranosyl)-5,6,7,8-tetrahydro-8R-hydroxy-6R-tridecylpyrimido[1,2-a]purine-10(3H)one and 3-(2-deoxy-*β*-d-erythro-pentofuranosyl)-5,6,7,8-tetrahydro-8S-hydroxy-6S-tridecylpyrimido[1,2-a]purine-10(3H)one [91]. Trans-2-hexadecenal forms Michael adduct with glutathione and with seven amino acids [92]. It also binds directly to Bax, leading to a conformational change in Bax, resulting in its activation, which induces apoptosis (Figure 1(b)) [24, 25]. It is not surprising that the long-chain aldehyde product interacts with many targets, as aldehydes are known to interact with proteins, nucleic acids, and carbohydrates. Ethanolamine derived from the SPL reaction was shown to be required for stationary phase differentiation and virulence in *Leishmania* [22]. Unlike other organisms that depend on sphingolipid biosynthesis and degradation for signaling and membrane structure/function, this organism depends on sphingolipid degradation primarily as its major route for ethanolamine synthesis. This raises the possibility that inhibiting SPL could be a useful strategy for the treatment of this parasitic disease.

## 4. Regulation of Lymphocyte Trafficking by S1P/S1PR1

S1P signaling has emerged as a central mediator of the trafficking of hematopoietic cells including lymphocytes, NK cells, neutrophils, dendritic cells (DCs), macrophages, hematopoietic progenitors, and mast cells [93–102]. Most evidence points to the role of S1P signaling in the control of hematopoietic cell egress from bone marrow, tissues, and lymphoid organs. However, there are instances where S1P signaling also regulates the entry of cells into the parenchyma, such as T cell entry into inflamed tissues [103]. S1PRs are expressed in all recognized immune cell types, although each cell type expresses only a subset of S1PRs. S1PR1 is expressed in most immune cells, whereas S1PR5 is expressed primarily by DCs and NK cells. S1PRs appear to have unique cell-specific functions. S1PR1 is most widely recognized for its crucial role in T and B lymphocyte trafficking, including the egress of mature T cells from the thymus and peripheral lymphoid organs [26].

### 4.1. S1PR1 Signaling and Recycling in T Cell Egress

**Figure 3:**
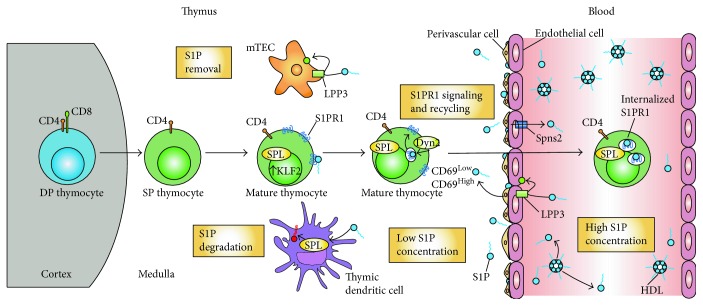
Egress of mature thymocytes from the thymus. Double-positive (DP) CD4^+^ and CD8^+^ thymocytes migrate from the thymic cortex to the medulla, where they differentiate into CD4^+^ and CD8^+^ single-positive (SP) thymocytes. As these cells mature, they express S1P lyase (SPL) and the transcriptional factor KLF2. The latter leads to increase transcription of its target gene, *s1pr1*, resulting in a high expression of surface S1PR1. Perivascular and endothelial cells in the corticomedullary region secrete S1P. Thymic dendritic and medullary thymic epithelial cells remove extracellular S1P by irreversible degradation or dephosphorylation to sphingosine, respectively. The resulting small S1P gradient interacts with the S1PR1 on the mature thymocytes. Prior to exit, S1PR1 receptors undergo multiple cycles of internalization to finally exit into the blood where there is a high S1P concentration. The high S1P concentration leads to S1PR1 permanent internalization and degradation.

Early thymic progenitor cells originating in the bone marrow enter the thymus at the corticomedullary junction and move into the cortex where they differentiate into T cell receptor- (TCR-) expressing CD4^+^CD8^+^ double-positive (DP) thymocytes (Figure 3). DP thymocytes undergo positive selection, a process in which thymocytes with inefficient T cell receptors are culled. Surviving thymocytes differentiate into semimature CD4 or CD8 single-positive (SP) cells that express CCR7. This population migrates to the medulla to undergo negative selection, a process in which thymocytes with self-recognizing T cell receptors are culled [104, 105]. The small number of semimature cells that have survived both positive and negative selections upregulates the transcription factor Krüppel-like factor 2 (KLF2), which promotes the transcription of *S1pr1* as well as other genes involved in homing, including CD62L [106, 107]. *Klf2* itself is also transcriptionally regulated by several factors including Foxo1 and PI3K/Akt [108, 109]. Activation of cell surface-localized S1PR1 by S1P enables the mature T cells to egress the thymus and enter the circulation via blood vessels located at the corticomedullary junction (CMJ) [3, 110]. Binding of S1P to S1PR1 on mature T cells activates RhoA GTPase and induces polarization of the actin cytoskeleton, as well as integrin clustering and activation. Mst1 and Mst2 kinases regulate the activation of RhoA and the migratory responses of SP thymocytes [111, 112]. Mst1/Mst2 kinases appear to act downstream of S1PR1 and upstream of RhoA, as mice lacking both kinases exhibit a block in thymic egress and show defects in RhoA activation and polarization of the actin cytoskeleton [112].

*S1pr1* mRNA expression is upregulated as thymocytes mature. A 50-fold increase is observed between DP and SP stages, while a 30-fold increase has been noted between immature and mature SP stages [26]. A defect in T cell egress and a concomitant depletion of mature T cells in the periphery has been noted in mice lacking *S1pr1* or *Klf2* in T lymphocytes [26, 106, 113]. Conversely, in a gain-of-function approach, either premature expression of S1PR1 in immature thymocytes or S1PR1 expression in thymocytes lacking *Klf2* was sufficient to drive their egress from the thymus, confirming that *S1pr1* is the main target of KLF2 needed for thymic egress [98].

Membrane expression of S1PR1 is negatively regulated by CD69, a lymphocyte activation marker. CD69 directly interacts with S1PR1 in a conformation that mimics some aspects of the ligand-bound state and promotes internalization and degradation [114, 115]. Mature SP cells express intermediate levels of CD69, but S1P signaling causes rapid downmodulation of surface CD69 [116]. Surface expression of S1PR1 on T cells is exquisitely sensitive to exposure to S1P. Nanomolar concentrations of S1P are sufficient to induce S1PR1 internalization. In response to persistent S1P signal, S1PR1 present on the T cell plasma membrane is phosphorylated by GPCR kinase-2 (GRK2) [117, 118]. *Grk2*-deficient T cells are resistant to S1P-mediated S1PR1 downmodulation even at micromolar concentrations [118]. S1PR1 phosphorylation recruits *β*-arrestins that dissociate the receptor from heterotrimeric G proteins, leading to receptor desensitization by terminating the signaling cascade. Recruitment of arrestins also causes S1PR1 internalization via clathrin-mediated endocytosis. Internalized S1PR1 can be degraded or recycled back to the cell surface. Binding of S1P to S1PR1 triggers activation of G proteins, thereby leading to effector function, but it also leads to receptor endocytosis [119, 120]. Receptor internalization through endocytosis and subsequent recycling is thought to be essential for restoring the responsiveness of S1PR1 [121, 122].

It has been proposed that the S1PR1 receptor desensitization (termination of S1P signaling) occurs via a GRK2-mediated pathway, whereas resensitization (recycling of S1PR1) occurs via dynamin 2-dependent endocytosis [123]. The *in vivo* response of S1PR1 appears to vary in different contexts, namely, in the presence of different concentrations of S1P. In an S1P-high environment such as the blood, receptor internalization is followed by its degradation, thereby terminating S1PR1 signaling and resulting in permanent desensitization. In contrast, in an S1P-low environment such as exit sites in the thymus, S1PR1 internalization does not trigger its degradation but instead results in continuous signaling via receptor recycling, ultimately facilitating T cell egress [123]. Both of these pathways are reciprocally regulated by GRK2 and a GTPase, dynamin 2. Although dynamin 2 is not required for the termination of S1PR1 signaling, dynamin-dependent endocytosis is required for S1PR1 resensitization in T lymphocytes. Dynamin 2 may help in maintaining sustained S1P-S1PR1 signaling in low S1P environments including the thymic CMJ, where T cells egress into the blood [123]. Mice lacking T cell dynamin 2 accumulate mature T cells in the thymus and exhibit profound lymphopenia [123]. Gatfield et al. [124] have shown that SPL is important for S1PR1 recycling in human umbilical vein endothelial cells by removing S1P from the receptor. Thus, SPL intrinsic to T cells may also be required for S1PR1 recycling and sustained S1P/S1PR1 signaling in mature T cells.

### 4.2. S1P Export and Transport

Erythrocytes and platelets are major contributors to blood S1P, as both of these cell types are exposed to circulating sphingosine, contain SphKs activity, and lack most of the S1P-degrading enzymes [30, 62]. Circulating S1P is cleared rapidly; the half-life of albumin-bound S1P in blood is ~17 min. S1P in the lymph is produced by radiation-resistant cells of nonhematopoietic origin, presumably lymphatic endothelial cells [125–127]. Mice lacking both isoforms of SphK in hematopoietic cells have undetectable S1P levels in plasma and lymph and exhibit a block in T cell egress from the thymus and lymph nodes [127]. Restoration of S1P content in the plasma of *Sphk* knockout mice (by infusion of erythrocytes from wild type-mice) rescued their lymphopenic phenotype by permitting thymic egress. These experiments confirm the requirement of plasma S1P for thymic egress [127].

After synthesis, S1P can be exported from the cell (Figure 2). S1P is an amphipathic molecule that requires facilitated export. The ATP-binding cassette (ABC) family of transporters including ABCA1, ABCC1, and ABCG2 has been shown to export S1P [128–130]. Erythrocytes and endothelial cells export S1P constitutively in a stimulus-independent manner. In contrast, mast cells and platelets secrete S1P in a stimulus-dependent manner and require the activity of ABCC1- and ABCA-like transporters, respectively. Another important regulator of extracellular S1P is the transporter Spns2, which has been shown to control S1P levels in the plasma and lymph by regulating S1P release from endothelial and lymphoendothelial cells [54, 131–133]. Global *Spns2* KO mice phenocopy *S1pr1* KO mice and exhibit a block in thymic egress and lymphopenia. Furthermore, Spns2-mediated export of S1P from endothelial cells is required for producing the S1P gradients required for the egress of B and T lymphocytes from the bone marrow and thymus, respectively [134, 135]. Once in the extracellular space, S1P can bind and activate the S1PRs present on neighboring cells (paracrine action) or on the same cell (autocrine action). The latter mechanism is also known as “inside-out signaling” [35].

In the blood, most of the plasma S1P is transported bound to high-density lipoprotein (HDL) (50–60%). The remaining part binds to albumin (30–40%), LDLs (∼8%), and very low-density lipoproteins (2-3%). Apo M (ApoM), a lipocalin protein, is the carrier of S1P in HDL (Figure 2). ApoM acts as an S1P chaperone and may additionally protect S1P from degradation and facilitate its presentation to receptors [125, 136]. Albumin-bound S1P and free S1P are susceptible to degradation, whereas HDL-bound S1P is more stable [125]. Levels of free S1P may be at least 40 times lower than the total S1P concentrations [125]. Many of the beneficial effects of HDL have been ascribed to its ApoM-S1P component [137].

### 4.3. Role of the S1P Gradient in Thymic Egress

Any chemotactic signal requires the presence of a chemical gradient in the local environment and a cellular receptor that senses that chemical gradient and stimulates reorganization of the cellular cytoskeleton, enabling movement toward or away from the gradient. A requirement for SPL and an S1P gradient in regulating T cell egress was first revealed by Schwab and colleagues [14]. They showed that pharmacological inhibition or genetic disruption of SPL causes ~1000-fold elevation in thymic S1P levels and disturbs the S1P gradient between parenchyma and the exit site, leading to a block in T cell egress [14, 70]. Immunosuppressant drugs such as FTY720 and THI work precisely by disrupting S1P signaling [14, 28, 138, 139]. FTY720 is a prodrug that is phosphorylated *in vivo* by SphK2 to become the active drug. The phosphorylated form, which is an analog of S1P, behaves as a super ligand; it tightly engages and sequesters S1PR1, 3, 4, and 5, thus inhibiting any downstream signaling. THI, on the other hand, inhibits SPL and prevents S1P degradation, thereby leading to increases in intracellular and extracellular S1P levels (Figure 2) [14]. Both drugs prevent the exit of T and B cells from secondary lymphoid organs and the thymus [28, 138, 139].

The S1P signal for T cell egress is produced on the front end via the synthesis and export of S1P by neural crest-derived pericytes surrounding thymic vascular endothelial cells [98]. We have recently shown that thymic dendritic cells (DCs), located at the CMJ, are responsible for maintaining the gradient at the back end, that is, by continuously degrading S1P in the thymic parenchyma, as described in detail below [140]. Endothelial cells also contribute to the S1P gradient by transporting S1P into the extracellular space via the Spns2 (Figure 2) [134]. Interestingly, endothelial cells may regulate the S1P gradient by removing extracellular S1P via dephosphorization through LPP3 [45]. Medullary thymic epithelial cells (TECs) described below also contribute to the low thymic S1P levels by transiently removing S1P through LPP3 (Figure 3).

### 4.4. Dendritic Cell SPL Generates the Chemotactic Gradient Essential for Thymic Egress

In the thymic tissue, SPL is predominantly expressed in stromal cells called TECs [61, 141]. To ascertain the role that SPL in the TEC compartment might play in regulating T cell egress, we used cre/lox technology to generate mice in which *SGPL1* is disrupted only in TECs. Surprisingly, we found that transgenic mice lacking TEC SPL exhibit normal T cell development, normal thymic S1P levels, appropriate levels of S1PR1 expression on mature T cells, and normal T cell egress [140]. Thus, despite the high SPL content in TECs, SPL activity intrinsic to TECs does not contribute appreciably to T cell egress.

Besides TECs, the thymus is largely comprised of endothelial and hematopoietic cells. To determine if SPL expression in endothelial and/or hematopoietic cell compartments is required for thymic egress, transgenic mice lacking SPL in these compartments (SPL^Mx1KO^ mice) were generated. When these mice are induced with polyinosinic:polycytidylic acid, *SGPL1* is disrupted in their spleen, blood, and liver. Deletion of SPL in hematopoietic and endothelial compartments using this strategy resulted in a twofold increase in CD4SP and CD8SP, as well as a fivefold increase in mature CD4SP and mature CD8SP T cells in the thymus. A concomitant depletion of CD4^+^ and CD8^+^ T cells was noted in the blood, lymph, spleen, and mesenteric lymph nodes. SPL deletion in hematopoietic and endothelial cells led to a marked increase in thymic S1P levels, accompanied by a decrease in the surface expression of S1PR1 in mature CD4SP cells [140]. These findings indicated that SPL is required in hematopoietic cells, endothelial cells, or both to establish the S1P gradient and to facilitate egress of mature T cells. Colocalization studies revealed that SPL is expressed in endothelial cells. We disrupted *SGPL1* in vascular and lymphatic endothelial cells through targeted recombination, and the resulting mice exhibited normal thymic egress and normal numbers of lymphocytes in the blood and secondary lymphoid organs. Furthermore, we did not observe significant differences in thymic S1P levels or surface expression of S1PR1 on mature CD4SP T cells between mice lacking endothelial SPL and control mice [140].

To confirm that SPL is required in the hematopoietic cell compartment to support T cell egress, we performed bone marrow (BM) reconstitution experiments. When irradiated SPL^Mx1KO^ mice were transplanted with the BM from wild-type mice, their thymic egress block was relieved, and they were no longer lymphopenic. Conversely, BM transplantation from SPL^Mx1KO^ mice to wild-type mice recapitulated the thymic egress defect. These data demonstrated that SPL expression in a cell type of hematopoietic origin must be required for egress [140].

To determine if SPL activity intrinsic to T cells enables their egress, we disrupted *SGPL1* in thymocytes at the DP stage. *SGPL1* disruption in T cells resulted in modest accumulation of T cells in the thymus and an increase in thymic S1P levels but did not phenocopy the severe thymic egress defect and peripheral lymphopenia observed in SPL^Mx1KO^ mice [140]. This suggested that T cell SPL contributed to their egress but was not the primary compartment in which SPL generates the S1P gradient. Besides thymocytes, three other hematopoietic cell types are present in the thymus, namely, B cells, macrophages, and DCs. SPL deletion in macrophages and B cells resulted in no effect on thymic S1P levels, S1PR1 expression, or T cell egress, suggesting that SPL in these cells is not required for the maintenance of the S1P gradient.

DCs are found in the thymic medulla and at the CMJ [98, 142]. We observed SPL expression in thymic DCs. To investigate whether DC SPL was required for egress, SPL^DCKO^ mice lacking SPL specifically in DCs were generated. SPL^DCKO^ mice exhibited prominent retention of SP cells and mature T cells in the thymus, were lymphopenic, and exhibited markedly high thymic S1P levels. Further, their mature T cells showed a decrease in surface expression of S1PR1 [140]. In a rescue experiment, mice lacking SPL in DCs were injected with wild-type DCs. These DCs with normal amounts of SPL homed to the thymus of recipient mice and restored lymphocyte egress [140]. These surprising cumulative observations show that thymic DCs, which constitute only a small percentage of thymic stromal cells, are the main metabolic gatekeepers of thymocyte egress.

To ascertain how DCs lower extracellular S1P levels to produce the gradient, we exposed bone barrow-derived DCs (BMDCs) to radiolabeled S1P *in vitro*. BMDCs could take up extracellular S1P in an S1PR-dependent manner that was blocked by treatment with FTY720. Collectively, these findings demonstrate that DCs play a central role in thymic egress by producing the S1P chemotactic gradient at the CMJ. Prior to this study, thymic DCs were recognized primarily for their role in negative selection and central tolerance. This is the first time thymic DCs have been shown to contribute to thymocyte egress by generating a localized S1P gradient (Figure 3).

One important question raised by our findings is whether DCs circulating throughout the body modulate their SPL expression or activity in response to environmental conditions such as infection, inflammation, and exposure to toxic stimuli. If so, can those DCs influence thymic output in response to these events in the periphery? Other S1P-metabolizing enzymes such as LPPs and ceramide synthase 2 have also been shown to regulate thymic S1P levels and thereby influence T cell egress [45, 143]. Why are so many enzymes required to maintain the S1P gradient? Are there other localized gradients that are required to sustain T cell egress from the thymus? Is the S1P gradient dynamically regulated? New methodologies may be required to answer some of these questions. Fortunately, elegant systems including reporter mice have recently been developed to facilitate the detection of S1P signaling footprints and signaling in real time in murine tissues [144]. With this novel approach, it may be possible to assess whether S1P gradients are dynamically regulated within tissues, orchestrating the movements of many cells simultaneously.

## 5. S1P Signaling and Inflammation

**Figure 4:**
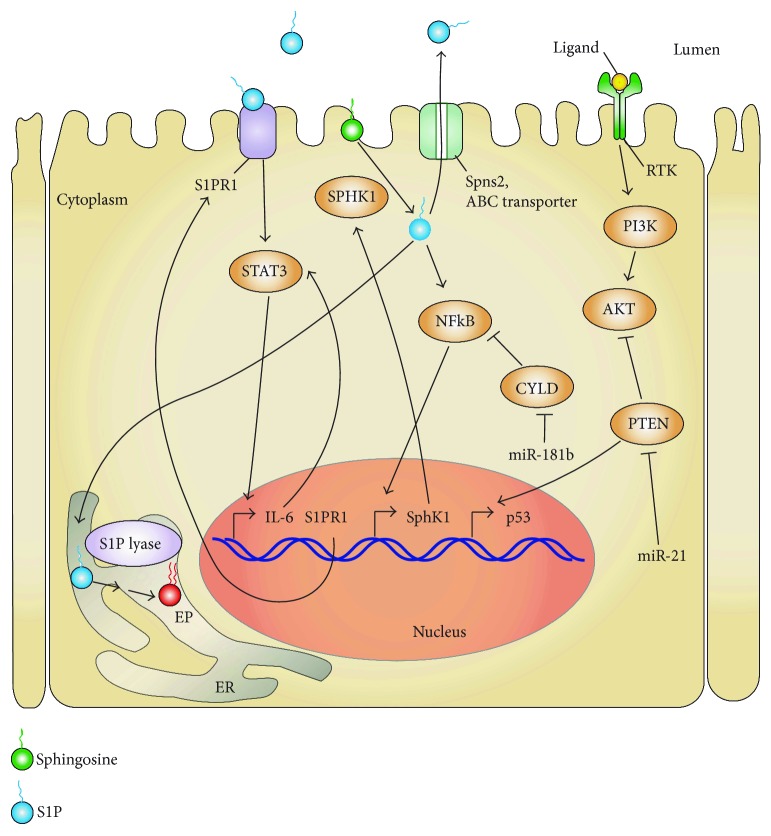
STAT3/S1P/S1PR1/SPHK1/SPL-positive feed-forward loop promoting CAC. In a chronic inflammatory milieu, STAT3 is persistently activated through two pathways. (1) SphK1 produces S1P which when kept intracellular can activate the NF*κ*B pathway leading to increased expression of SPHK1. (2) When S1P is exported out of the cells via the SPNS2 transporter, the binding of S1P to its S1PR1 activates STAT3. STAT3 upregulates IL-6 and S1PR1 expression as well as miR-181b1 and miR-21. miR-181b1 decreases the expression of the tumor-suppressor CYLD which results in derepression of the NF*κ*B pathway. miR-21 decreases the expression of PTEN which is an antioncogene negatively regulating the PI3K/AKT pathway and positively p53. All these pathways are interrelated in an amplification loop, permitting the persistent STAT3 activation, which is involved in cell transformation and tumor progression. Bent arrows indicate transcriptional regulation.

S1P has been implicated as a critical factor in the signaling and biological functions of inflammatory mediators such as lipopolysaccharide (LPS), tumor necrosis factor- (TNF-) *α*, and interleukin- (IL-) 1*β*. Through the activation of its receptor, TNF-*α* induces the recruitment of TNF receptor-associated factor 2 (TRAF2), an E3 ubiquitin ligase that activates I kappa B kinase proteins, which results in NF*κ*B signaling through the canonical pathway. Alvarez et al. demonstrated that intracellular S1P generated by SphK1 binds to TRAF2 and acts as its cofactor [145]. This study revealed the role of intracellular S1P generation in the activation of the canonical NF*κ*B signaling pathway. In a model of bronchial asthma, S1PR2 activation by S1P increased the expression of the chemokine (C-C motif) ligand- (CCL-) 3 through the activation of NF*κ*B and STAT3 signaling pathways [146]. Administration of JTE013, a specific S1PR2 inhibitor, reduced both NF*κ*B and STAT3 activations resulting in decreased CCL3 expression. STAT3 can be activated through S1PR1 signaling following S1P stimulation. We showed that when SPL is silenced in mouse embryonic fibroblasts, extracellular S1P activates STAT3 through an S1PR1-dependent pathway, resulting in decreased expression of the antioncogene CYLD, a negative regulator of NF*κ*B [147] (Figure 4). In a mouse model of chronic colon inflammation, Nguyen et al. demonstrated that the increased STAT3 signaling in epithelial cells under inflammatory conditions was a result of S1P-dependent activation of S1PR1 [148] (Figure 4). By activating epithelial STAT3, S1P signaling promoted higher expression of proinflammatory cytokines including IL-17 and IL-6 and decreased the expression of CCL19, CCL28, and RANTES, which are regulatory T cell (Treg) recruiters and, therefore, have anti-inflammatory functions. Flow cytometry analysis of the inflamed colon under these conditions showed increased recruitment of cytotoxic CD8^+^ T cells and reduced recruitment of Tregs [148]. CD8^+^ T cells are thought to play a key role in promoting tissue damage to the colonic epithelium and are associated with enhanced inflammation in patients suffering from IBD [149]. Tregs suppress the activity of CD8^+^ T cells by affecting granule exocytosis, thereby rendering T cells unable to kill their target cells [150]. This study illustrates the role of the S1P/S1PR1/STAT3 axis in the regulation of colitis. These examples demonstrate that S1P signaling contributes to the inflammatory processes mainly through the activation of two key proinflammatory transcription factors, NF*κ*B and STAT3.

### 5.1. SPL Regulation of Inflammatory Signaling

SPL is crucial for attenuating S1P signaling and its effect on inflammatory pathways. Mice with an inactive SPL gene (*SGPL1*) die soon after weaning and have high levels of circulating and tissue S1P leading to increased levels of proinflammatory cytokines (TNF, IFN-*γ*, MCP-1, and IL-6) and upregulation of genes associated with acute-phase response (SAA1, SAA3) in the liver [151]. Similarly, a role for SPL has been established in cardiomyocytes in a study of mice treated with the SPL inhibitor THI [152]. The mice treated with THI have higher levels of S1P in cardiac tissue resulting in aggravated pathological cardiac remodeling after myocardial infarction. In this context, S1P activation of S1PR1 activated both NF*κ*B and STAT3 pathways increasing the expression of proinflammatory cytokines TNF-*α* and IL-6, which could be reverted by blocking S1PR1 with the inhibitor W146. These studies highlight an anti-inflammatory role for SPL. However, a recent paper from Ebenezer et al. demonstrated a proinflammatory role for SPL [23]. In an *in vitro* model of LPS-induced lung inflammation, the authors demonstrated that the end products of S1P degradation by SPL (*trans*-2-hexadecenal and phosphoethanolamine) modulated the histone acetylation profile in a different way than that mediated by nuclear S1P produced by SphK2 [23]. Blocking SPL resulted in modified HDAC acetylation patterns and decreased secretion of proinflammatory cytokines normally induced following LPS treatment in lung endothelial cells. These results are in accordance with a paper by Zhao et al. showing that intratracheal instillation of LPS to mice increased the levels of SPL leading to decreased levels of S1P in the lung tissue which ultimately resulted in lung inflammation and injury. Inhibiting SPL with THI had a protective effect resulting in less lung inflammation and injury. The increased lung S1P level attenuated phosphorylation of p38-MAPK and I-*к*B, IL-6 secretion, and endothelial disruption normally attributed to LPS treatment [82].

These studies indicate that manipulating SPL and S1P for therapeutic purposes may represent some challenges. Both of these targets control cell survival, barrier function, epigenetic gene regulation, and inflammatory signals that may protect tissues and help maintain tissue homeostasis in the face of acute insults but may induce injury when sustained. Colitis and CAC are well-established examples of how tissue homeostasis can be lost in the face of chronic inflammatory signaling. The above findings highlight the need for further exploration of the best ways to target the S1P pathway therapeutically in a disease-dependent context.

### 5.2. S1P and Inflammation-Associated Cancer

Patients suffering from chronic inflammation and other autoimmune diseases are at higher risk of developing cancer than the general population [153]. The mechanism for this observation has not been fully elucidated, although many potential explanations have been proposed as elegantly reviewed by Elinav et al. [154]. It is thought that in the inflamed tissue, the production of reactive oxygen species by immune cells induces DNA damage. This insult is combined with an inflammatory milieu containing cytokines that stimulate cell survival and proliferation, primarily through the activation of the transcription factors NF*κ*B and STAT3. The activation of these two signaling hubs by S1P can result in a change in the cell's genetic program that facilitates cell transformation and the acquisition of the cancer phenotype. S1P levels are high in chronic inflammation, and several studies have confirmed the role of S1P in the development of cancer. Lee et al. showed a positive feed-forward loop implicating STAT3 and S1P/S1PR1 signaling pathways in carcinogenesis and metastasis using mice bearing urothelial carcinoma MB49 or melanoma B16 tumor cells [155] (Figure 4). The mutual coactivation of these pathways results in the persistent activation of STAT3 which promotes carcinogenesis by upregulating the expression of IL-6 and additional factors involved in tumor growth and metastasis. The most well-established examples of S1P's role in inflammation-associated cancer are in the context of CAC. Nguyen and colleagues demonstrated that when chronic inflammation is present, increased IL-6 expression leads to persistent activation of intestinal epithelial STAT3, which regulates S1P production through SphK1 [148] (Figure 4). As mentioned earlier, the resulting imbalance of cytotoxic CD8^+^ T cells versus Treg cells intensifies intestinal inflammation, thus promoting tumor progression and malignancy. Liang et al. also demonstrated that colonic inflammation could lead to CAC through a positive-feed forward loop linking persistent STAT3 activation and S1P/S1PR1 signaling [156] (Figure 4). Consistent with previous studies, they showed that SphK1 and S1PR1 expressions were increased in colitis and CAC, concomitant with an increased level of tissue S1P. They went further to show that both extracellular and intracellular S1P contributed to the development of tumorigenesis. They showed that extracellular S1P activated STAT3 through S1PR1 in a Src-dependent manner. By activating NF*κ*B, intracellular S1P resulted in increased expression of both IL-6 and TNF-*α*, which led to further STAT3 activation and activation of SphK1. In a CAC mouse model, treatment with FTY720 to block S1PR1 activation reduced both upregulations of S1PR1 and SphK1 in the carcinomas.

### 5.3. SPL Regulation of Inflammation-Associated Cancer

Studies from our group demonstrated that SPL expression is high in the intestinal epithelium but downregulated in murine adenomas and human colorectal cancers [11, 61]. This finding, combined with a growing appreciation for the key role S1P signaling plays in colon cancer [156–159], prompted us to investigate the potential effect of gut epithelial *SGPL1* disruption on the development of CAC. Toward that end, we generated an inducible intestinal epithelium-specific SPL KO mouse (SPL^GutKO^) using Cre/lox technology [10, 147]. SPL^GutKO^ mice showed an 8-fold increase in S1P levels in the small intestine and colon tissues, whereas plasma S1P levels were unchanged, demonstrating that the SPL^GutKO^ mouse is a good model system for testing the effects of localized S1P accumulation on intestinal biology. There were no obvious changes in gut epithelial architecture, weight, litter size, or life span in SPL^GutKO^ mice compared to “floxed” littermates lacking the Cre Tg used as controls. However, when SPL^GutKO^ mice were treated with dextran sodium sulfate (DSS) to induce a chemical colitis, they developed more severe colitis than the controls, with shortened colon length, increased disease activity, and higher levels of tissue S1P compared to DSS-treated controls. When SPL^GutKO^ mice were treated with both the carcinogen azoxymethane and DSS to induce CAC, they exhibited an increased susceptibility to CAC compared to controls [147]. The SPL^GutKO^ mice exhibited reduced survival and an increased intensity of disease. They also exhibited increased levels of plasma and tissue cytokines including TNF-*α*, IL-1*β*, and IL-6. Importantly, these mice also were found to have higher percentages of Th17 cells and increased number of macrophages than controls. Th17 cells and STAT3-mediated inflammation correlate with autoimmune diseases like experimental autoimmune encephalitis and colitis [160, 161]. In SPL^GutKO^ mice, the number of tumors per mouse was higher than in controls, whereas tumor size and grade were unaffected. However, using Ki67^+^ staining, we observed a higher number of proliferating cells in the region surrounding the tumors in the SPL^GutKO^ mice. This finding prompted us to hypothesize that SPL deletion promotes an early step of the carcinogenic process, such as cell transformation. Studies by Hua Yu and colleagues that had linked S1P to STAT3 signaling in 2010 prompted us to examine this pathway in SPL^GutKO^ mice (Figure 4). We found that STAT3 phosphorylation was increased in SPL^GutKO^ mice compared to control mice. In addition, the STAT3 target genes *c-Myc*, *Mcl-1*, and *S1pr1* as well as *Sphk1* were all increased in the nontumor colon tissue of SPL^GutKO^ mice compared to controls [147]. STAT3 is a transcription factor for multiple genes associated with inflammation and tumor progression, but it is also an important transcription factor for micro RNAs (miRNAs). Iliopoulos et al. demonstrated that persistent STAT3 activation leads to inflammation and cancer by increasing the expression of miR-181b1 and miR-21 which silence two tumor-suppressor genes *Cyld* and *Pten*, respectively [162]. PTEN is a phosphatase that acts by suppressing PI3K signaling, whereas CYLD is an ubiquitin ligase that acts by inhibiting NF*κ*B signaling. We found that expressions of miR-181b and miR-21 were elevated, whereas their target proteins CYLD and PTEN were diminished in SPL^GutKO^ mice colons compared to those of control mice. The increased susceptibility of SPL^GutKO^ mice to tumorigenesis was rescued by the administration of the STAT3 inhibitor NSC 74859. We then examined the impact of SPL silencing in mouse embryonic fibroblasts (MEFs) harboring either functional or nonfunctional STAT3. SPL silencing in MEFs resulted in an increase in the expression of the Spns2 and a concomitant increase in the extracellular levels of S1P. Moreover, MEFs lacking SPL proliferated faster than those with wild-type SPL and formed tumors when injected subcutaneously in NOD/SCID mice, but only in the presence of wild-type STAT3 and not in its absence. These results combined with our *in vivo* findings confirmed that SPL silencing renders cells susceptible to cell transformation in a STAT3-dependent manner [147].

Our findings raise additional questions that will require further study. What is the role of SPL contained within other immune cells that are implicated in the development of CAC? Why did we observe an increased number of macrophages in SPL^GutKO^ mice suffering from CAC? This is important, as tumor-associated macrophages are known to play a key role in maintaining the tumor microenvironment suitable for neoplastic transformation [163]. It will also be important to determine how the inflammatory milieu, oxidant stress, diet, and the microbiome affect SPL expression and activity, since gut epithelial SPL and these environmental factors all influence the susceptibility to colitis and CAC.

## 6. SPL as a Therapeutic Target for Inflammatory Diseases

In the past three decades, S1P signaling has been associated with numerous inflammatory diseases such as psoriasis, asthma, rheumatoid arthritis, multiple sclerosis, and inflammatory bowel disease [164]. As novel molecules targeting the sphingolipid pathway components are developed and proven efficacious in preclinical models of inflammatory diseases, as recently reviewed by Park and Im [165], they are moving forward into clinical trials in various disease contexts. In addition to S1P receptor agonist and antagonists [2], SPL inhibitors represent potential treatment options for inflammatory disorders such as multiple sclerosis, rheumatoid arthritis, and inflammatory bowel disease. SPL has a dual role in inflammation. Inhibition of SPL enzyme activity by a pharmacological agent for a short time could inhibit inflammation by blocking the entry of T lymphocytes to the site of injury/damage. In contrast, a sustained decrease in SPL enzyme activity or SPL deficiency will increase S1P levels and thereby may activate NF*κ*B and STAT3 transcription factors. Both of these transcription factors are known to induce the gene expression of proinflammatory cytokines.

Genetic evidence suggests that partial SPL inhibition may be beneficial in several inflammatory conditions. For example, partial SPL deficiency provides protection in EAE in mice [18]. SPL-deficient mice exhibit reduced migration of T cells into the CNS [18]. Thus, similar to FTY720, SPL inhibitors could have utility in the treatment of multiple sclerosis. THI, a component of caramel color III, inhibits SPL enzyme activity *in vivo* but not *in vitro*. Until recently, the mechanism by which THI inhibits SPL was not clear. Ohtoyo and colleagues revealed that orally delivered THI is metabolized *in vivo* by the gut microbiome [166]. They showed further that key metabolite of THI, which they synthesized and named A6770, is phosphorylated by cells and inhibits SPL *in vitro* and *in vivo* [166]. We have confirmed the activity of A6770 as an SPL inhibitor *in vitro*, as well as the loss of THI-mediated lymphopenia by pretreatment with antibiotics to deplete the gut microbiome (our unpublished observations). Lexicon Pharmaceutical Inc. has developed several inhibitors of SPL including LX2931, LX2932, and LX3305. These compounds, which are THI analogs, were shown to inhibit inflammatory responses in animal models of rheumatoid arthritis, multiple sclerosis, and transplantation [167]. Unfortunately, LX3305 did not show promising results in phase II clinical trial in rheumatoid arthritis patients receiving methotrexate [168]. However, considering that methotrexate is an antibiotic that interferes with bacteria's ability to synthesize folate, it is likely that this drug combination abrogated the impact of LX3305 on SPL and, consequently, on inflammation.

Several other examples of the impact of SPL inhibition on inflammation are instructive. A mouse model of cystic fibrosis exhibited reduced levels of S1P in the lungs. Oral treatment of these mice with LX2931 raised S1P levels in the tissues and reduced lung inflammation by decreasing the levels of inflammatory cytokines [80]. Sepsis is a systemic inflammatory response to pathogens and a leading cause of hospital-related mortality worldwide. Administration of 4-deoxypyridoxine (DOP), a nonselective inhibitor of SPL, ameliorated morbidity, improved recovery from sepsis in surviving mice, and reduced sepsis-induced hypothermia. Treated mice also showed reduced bacterial burden in the liver, although not in the blood [169]. NK cells have been shown to localize in the medullary region of lymph nodes, and an S1P gradient is required for the proper localization of NK cells to this site [170]. This raises the possibility that SPL could be targeted in cancer for control NK-cell mediated tumor cytotoxicity. In fact, a recent study supports this notion [171]. Complete deficiency of SPL could enhance transformation and cause severe complications similar to those observed in patients with *SGPL1* mutations. However, partial SPL inhibition with small molecule inhibitors may be beneficial in certain contexts, especially using short-term or periodic administration.

Identification of SPL as a promising therapeutic target for inflammatory diseases has intensified interest in the identification of small molecules that modulate its activity. To facilitate testing of a large number of compounds, newer high throughput methods are being developed. Kashem et al. developed a high-throughput scintillation proximity assay to screen SPL inhibitors [172]. The major limitation in the development of novel SPL inhibitors is the lack of a crystal structure for human SPL. In order to overcome this problem, bacterial SPL from *Symbiobacterium thermophilum* has been mutated, which would facilitate structure-based designing of novel SPL inhibitors [173]. Recombinant StSPL has been shown to degrade extracellular S1P *in vivo* [60]. Thus, StSPL may be used as an enzyme replacement therapy for patients with SPL deficiency, suffering from nephrotic syndrome, adrenal insufficiency, and neurological problems, and for patients with fibrotic kidney disease.

## 7. Conclusion

In conclusion, SPL is a ubiquitously expressed enzyme that is essential for maintaining S1P chemotactic gradients that enable T cell egress and other aspects of hematopoietic cell trafficking. In addition, SPL appears to regulate S1P/STAT3 signaling in the context of intestinal inflammation, colitis, and the development of CAC. In addition to its influence on S1P signaling, SPL generates unique products that exhibit biological activities and control upstream sphingolipid intermediates, since SPL represents the only exit point for the entire sphingolipid degradative pathway. As such, SPL modulation has pleiotropic effects, and mutations in human *SGPL1* are associated with a variety of disease states including but not limited to immunodeficiency. Future studies involving SPL-deficient patients and their biological samples and utilizing SPL inhibitors, model organisms, and global, partial, targeted, and inducible mouse models of SPL deficiency will help clarify the role of SPL in physiology, disease, and therapeutics.

## Abbreviations

ABC: ATP-binding cassette
ApoM: Apolipoprotein M
BM: Bone marrow
BMDC: Bone marrow-derived dendritic cells
CAC: Colitis-associated cancer
CMJ: Corticomedullary junction
CNS: Central nervous system
DC: Dendritic cell
DP: Double-positive
DSS: Dextran sodium sulfate
EAE: Experimental autoimmune encephalomyelitis
ER: Endoplasmic reticulum
GRK: G protein-coupled receptor kinase
HSC: Hematopoietic stem cell
IBD: Inflammatory bowel disease
IL: Interleukin
KLF: Krüppel-like factor
KO: Knockout
LPP: Lipid phosphate phosphatase
LPS: Lipopolysaccharide
MAM: Mitochondria-associated membrane
MEF: Mouse embryonic fibroblast
miR: MicroRNA
NF*κ*B: Nuclear factor kappa B
NK: Natural killer
PLP: Pyridoxal phosphate
S1P: Sphingosine-1-phosphate
SPL: Sphingosine-1-phosphate lyase
S1PR: Sphingosine-1-phosphate receptor
STAT3: Signal transducer and activator of transcription 3
SphK: Sphingosine kinase
SC: Satellite cell
SRNS: Steroid-resistant nephrotic syndrome
SP: Single-positive
TEC: Thymic epithelial cell
THI: 2-Acetyl-4-(tetrahydroxybutyl) imidazole
TNF: Tumor necrosis factor
TRAF2: Tumor necrosis factor-associated factor 2
Treg: Regulatory T cell.
